# Molecular Phylogeny Reveals High Diversity, Geographic Structure and Limited Ranges in Neotenic Net-Winged Beetles *Platerodrilus* (Coleoptera: Lycidae)

**DOI:** 10.1371/journal.pone.0123855

**Published:** 2015-04-28

**Authors:** Michal Masek, Vaclav Palata, Timothy C. Bray, Ladislav Bocak

**Affiliations:** Department of Zoology, Faculty of Science, Palacky University, 17. listopadu 50, 771 46, Olomouc, Czech Republic; Universidad Nacional Autonoma de Mexico, MEXICO

## Abstract

The neotenic *Platerodrilus* net-winged beetles have strongly modified development where females do not pupate and retain larval morphology when sexually mature. As a result, dispersal propensity of females is extremely low and the lineage can be used for reconstruction of ancient dispersal and vicariance patterns and identification of centres of diversity. We identified three deep lineages in *Platerodrilus* occurring predominantly in (1) Borneo and the Philippines, (2) continental Asia, and (3) Sumatra, the Malay Peninsula and Java. We document limited ranges of all species of *Platerodrilus* and complete species level turnover between the Sunda Islands and even between individual mountain regions in Sumatra. Few dispersal events were recovered among the major geographical regions despite long evolutionary history of occurrence; all of them were dated at the early phase of *Platerodrilus* diversification up to the end of Miocene and no exchange of island faunas was identified during the Pliocene and Pleistocene despite the frequently exposed Sunda Shelf as sea levels fluctuated with each glacial cycle. We observed high diversity in the regions with persisting humid tropical forests during cool periods. The origins of multiple species were inferred in Sumatra soon after the island emerged and the mountain range uplifted 15 million years ago with the speciation rate lower since then. We suppose that the extremely low dispersal propensity makes *Platerodrilus* a valuable indicator of uninterrupted persistence of rainforests over a long time span. Additionally, if the diversity of these neotenic lineages is to be protected, a high dense system of protected areas would be necessary.

## Introduction

**Fig 1:**
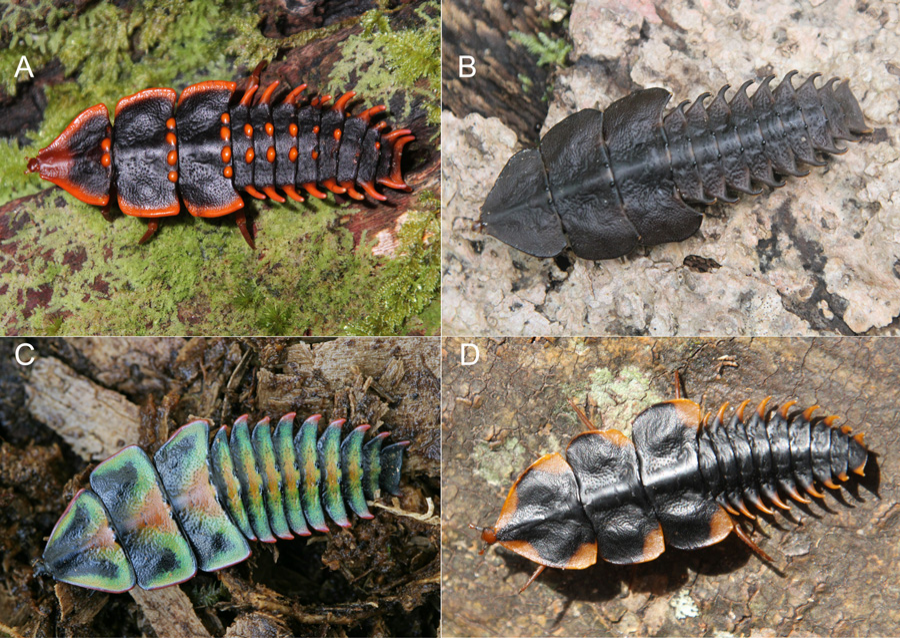
Female larvae, late instars. A-C *Platerodrilus*, Bornean clade; A—from Borneo, Sabah, Mt. Kinabalu, B—Borneo, Sabah, Poring, C—Mindanao, New Bataan; D—*Macrolibnetis*, Malaysia, Pahang, Cameron Highlands.

The lyropaeine net-winged beetles (Lycidae) are a rare example of developmental modifications between sexes [1, 2]. The males undergo complete metamorphosis, passing through the pupal stage, to become fully winged adults, with body length varying from 1.3–10 mm [3]. In contrast, the females remain larviform when sexually mature and differ from larvae only in the structure of cuticle and opened sexual tract (Fig 1) [4, 5]. The neotenic development of females has several macroevolutionary consequences. First of all, the winglessness results in the very limited dispersal propensity of all net-winged beetles with larviform females. Males, although winged, fly only in short distances within the lowest strata of the tropical rainforests, when searching for females (personal observation). Further some lyropaeine beetles have very large bodied females unknown in non-neotenic lineages of net-winged beetles ("trilobite larvae", body length 30–70 mm, Fig 1). These females live for several years in the larval stage and produce a relatively low number of large eggs from which hatch larvae comparable in size to adult males [5]. Such a reproductive strategy is energetically costly and was hypothesized as a case of K-strategy [1]. The high female investment in offspring has also been similarly predicted also for other neotenics [6]. Unlike females, the conspecific males often have a very small body (6–10 mm in *Platerodrilus*, but slightly over 1 mm in *Alyculus*), live only shortly in the adult stage [5], and invest a minimum energy in offspring compared to females. Apparently, the different selective regime affects the males and results in very disparate adult morphology. Although information on these beetles remains incomplete and females are unknown for most species and genera, the significant morphological differences among large-bodied females were proved in *Platerodrilus* [4, 5], *Macrolibnetis* [7] and *Lyropaeus* [2] and the previous studies support the parallel origins of extremely large females.

**Fig 2:**
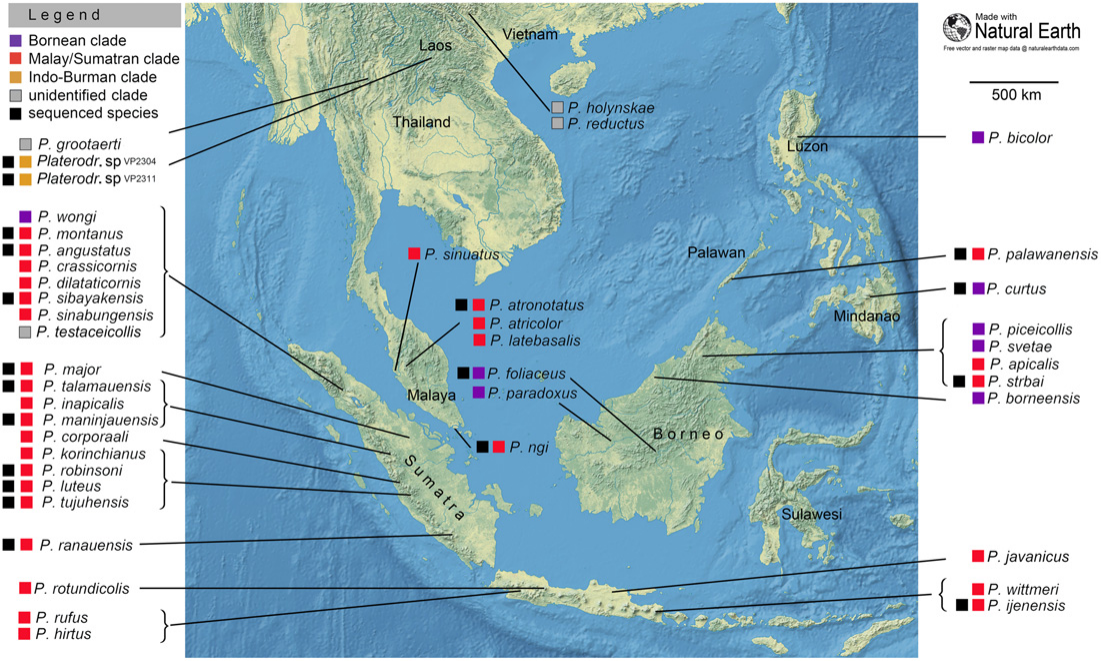
Distribution of *Platerodrilus*. The taxa were assigned to clades by the phylogenetic analysis and/or morphological similarity. *P*. *testaceicollis* Pic, 1921 is known in a damaged holotype and no diagnostic characters are available to evaluate its relationships.

Forty-two species of *Platerodrilus* are known and their ranges are limited to three major biodiversity hotspots [8]: Indo-Burma, Sundaland and the Philippines including Palawan [3]. *Platerodrilus* do not occur in continental China (except the southernmost part of Yunnan along Lao and Burman borders) or in the southern part of the Indian subcontinent (Fig 2).

The majority of elateroid beetles are winged and quite effective at dispersal. On the other hand, there are multiple lineages within Elateroidea with wingless to larviform females. These lineages, e.g. Omethidae: Telegeusinae, multiple lineages of Lampyridae, all Omalisidae and Plastoceridae, Elateridae: Cebrioninae, Agrypninae: Drilini and others, usually share with their close relatives the biology and differ only in an incomplete metamorphosis. The lycid neotenic females remain completely larviform, their dispersal ability is extremely low and they heavily depend on humid rainforests as their larvae need constant access to liquid food and even the winged males do not fly effectively. Additionally, they are relatively species rich and previous studies have suggested their persistence in stable habitats [3, 5]. Therefore, these neotenic beetles can be used as a model group for evolutionary and zoogeographical studies and their presence and *in situ* diversification can identify regions with uninterrupted persistence of the rainforests during climatic fluctuations. Herein, we present the multi-marker molecular phylogeny of *Platerodrilus* and reconstruct the ancestral range and dispersal routes of *Platerodrilus* in the Oriental region. The aim of this study is to recover the diversification history of *Platerodrilus* and define ranges with occurrence of ancient lineages of the neotenic beetles in South East Asia. We suggest that these are biodiverse regions worthy of protection and whose constituent species would be in danger of extinction should the habitats be destroyed.

## Material and Methods

The specimens were preserved in 96% ethanol in the field and stored in a freezer until DNA was isolated. The sampled localities included the Kinabalu and Emas National Parks (Access Licence JMK/MBS.1000-2/2(110) issued by the Sabah Biodiversity Council, and Permit Penyelidikan No TS/PTD/5/4Jld48(41) by Lembaga Pemegang Amanah Taman-Taman). Other material was collected outside protected areas and species included in the research are not protected by international or national laws. The sequenced specimens are deposited in the voucher collection of the Laboratory of Molecular Systematics, Faculty of Science, Olomouc, Czech Republic, only the voucher specimen of *P*. *ngi* is deposited the Zoological Reference Collection, Raffles Museum of Biodiversity Research, National University of Singapore. The voucher numbers provide access to all sequenced DNA fragments deposited in GenBank (S1 Table).

### Laboratory methods and phylogenetic analyses

DNA was extracted following the standard protocol with Wizard SV96 kit (Promega) from the flight muscles and larval thoracic segments. Extraction yield was measured using a NanoDrop-1000 Spectrophotometer. PCR was performed in a 50 μl reaction volume with 0.5 U Taq polymerase, 1 mM MgCl_2_, 50 mM of dNTPs, 0.2 mM of each primer, and typically 30 ng of template. Cycle conditions were generally 2 min at 94°C, 30–60 sec at 94°C, 30–60 sec at 45–52°C, 1–2 min at 72°C (repeated steps 2–4 for 35–40 cycles), and 10 min at 72°C. Two nuclear DNA markers were included in the study: the complete 18S rRNA (~1900 bp), the D2 region of the 28S rRNA (630 bp). Three fragments of the mitochondrial genome were sequenced: the 800 bp *cox1* 3′ region of *cox1*, the 1200 bp fragment of *nad5* and adjacent *tRNA*-*Phe*, *tRNA*-*Glu*, *tRNA*-*Ser* (the whole fragment referred as *nad5* further), and a fragment of *rrnL* (530 bp). The used primers are listed in S3 Table. PCR products were purified using PCRu96 Plates (Millipore Inc.) and sequenced by an ABI 3130 automated sequencer using the Big Dye Terminator Cycle Sequencing Kit 1.1.

### Sequence handling and phylogenetic analyses

Sequences were edited using the Sequencher 4.8 software package (Gene Codes Corp.). The protein-coding markers, *i*.*e*., *nad5* and *cox1* were aligned by ClustalW 1.83 [9] under default settings. Length variable fragments, *i*.*e*., 18S, 28S rRNA, *rrnL*, and tRNA mtDNA fragments, were separately aligned using ClustalW 1.83, BlastAlign 1.2 [10] under default parameters, and Muscle 3.6 [11] under the gap opening parameter 2600 and gap extension parameter 240. All DNA fragments were concatenated for final analyses. Multiple alignment methods were chosen to test the impact of various procedures on the tree topology. Phylogenetic analyses were carried out under the likelihood criterion (ML) using RAxML 7.2.3 [12] and bootstrap support of branches (BS) were assessed by analyzing 100 pseudoreplicates. All genes and codon positions in the protein coding fragments were partitioned under the models proposed by jModelTest 0.1.1 [13]. The dataset was additionally analyzed using the Bayesian inference (BI) implemented in MrBayes 3.2.2 [14]. The Markov Chain Monte Carlo (MCMC) was set for independent variability of parameters in individual coding and non-coding genes under the GTR+I+G model. Two runs, each with four chains ran simultaneously for 40 x 10^6^ generations, with trees being sampled every 1000^th^ generation, all fragments were partitioned and unlinked. The results were evaluated in Tracer 1.6 [15]. The first 2 x 10^6^ trees of the first run and 16 x 10^6^ trees of the second run were discarded as burn-in and posterior probabilities (PP) at nodes were determined from the remaining trees in the analysis of the Muscle alignment, 5 x 10^6^ and 5 x 10^6^ generations were discarded as burn-in in analysis of the BlastAlign matrix and 21 x 10^6^ and 12 x 10^6^ generations in the analysis of the Clustal alignment. The convergence of all parameters was assessed when runs were combined and the effective sample size (ESS) was over 1000 in all analyses. We used the MP-based statistical dispersal-vicariance analysis implemented in RASP 2.1 [16] for an ancestral range reconstruction on the best ML tree inferred from the Muscle alignment. We coded six geographical regions: (1) Continental Asia north of Isthmus Kra represented by Lao and Thai species (2) Peninsular Malaysia, (3) Sumatra, (4) Java, (5) Borneo and (6) the Philippines. Two regions were simultaneously allowed at node during optimization.

The relative age of nodes was estimated using penalized likelihood and cross-validation analysis as implemented in r8s 1.71 [17]. The age of the in-group was arbitrary set to 100 as no fossils are available for calibration. We used previously inferred age of *Platerodrilus* [1] for an approximate estimation of the absolute age of critical dispersal events and speciation in Sumatra. Branch lengths were optimized on the preferred tree topology and a wide range of smoothing parameters were tested before final analysis. Sampling intervals for inferred divergences were obtained from 100 bootstrap pseudoreplicates as described in the r8s manual. The lineage-through-time (ltt) plot for *Platerodrilus* was constructed from the inferred normalized tree using the Phytools package 0.4–05 in R ([18]; http://r-forge.r-project.org/projects/splits/).

Additionally, we estimated the time to the most recent common ancestor for *Platerodrilus* using a Bayesian approach implemented in Beast 1.8.1 [19]. The analysis was performed using the Muscle alignment and a GTR+I+G model as given by the AICc criterion in jModelTest 3.7 [13], a relaxed molecular clock and an uncorrelated lognormal model of rate variation among branches. The *Platerodrilus* clade was fixed to have an age of 1.0 and subsequently calibrated with previously hypothesized age [1]. The data were partitioned according to the genes and codon positions in the protein coding genes, with each partition allowed independent parameters. Altogether 5 x 10^7^ generations were run, trees sampled every 10,000 generations and 5 x 10^6^ generations were erased as pre-stationary stage. Convergence and ESS were assessed in Tracer 1.5 [15].

There are no fossils available for more accurate dating of the tree and we depend on the previous estimation of origin of major net-winged beetle lineages [1]. Therefore, we specifically ask about the origin of the Philippine fauna (ancient colonization in the Oligocene / Lower Miocene 20–35 mya versus recent dispersal history during low sea stands in the Upper Pliocene and Pleistocene) and putative accelerated speciation in Sumatra when it was established in the present form about 15 mya [20].

## Results

### Sequence variation

We concatenated the DNA sequences of five fragments: 18S rRNA (78 specimens), 28S rRNA (74 spec.), *rrnL* (75 spec., published by Masek and Bocak [3]), *cox1* (60 spec.) and *nad5* mtDNA (61 spec.). Amplification problems were commonly encountered when tissue was taken from larvae and as a result only 78.6% of markers were available for *Platerodrilus*. The complete dataset of aligned 18S, 28S, *rrnL*, *cox1*, and *nad5* DNA fragments contained 5086–5274 homologous positions depending on the applied alignment procedure (Table 1); 1727–1816 characters were parsimony informative. The aligned 18S fragment contained 1873–1883 positions, (7.6% of the parsimony informative characters), 28S 637–641 positions (3.8%), *rrnL* 530–723 positions (14.5%), *cox1* 790 positions (24.7%), and *nad5* 1237–1284 positions (47.0%).

**Table 1:** The length of DNA fragments and the numbers of informative characters in datasets (gaps considered as the 5^th^ character).

Datasets		All data	18S rRNA	28S rRNA	*rrnL*	*cox1*	*nad5-tRNAs*
	# of spec.	83	78	74	75	60	61
ClustalW	# of char.	5087	1875	637	530	790	1255
	parsimony informative	1816	138	67	267	447	897
BlastAlign	# of char.	5274	1883	641	723	790	1237
	parsimony informative	1727	134	73	253	447	820
Muscle	# of char.	5116	1873	638	531	790	1284
	parsimony informative	1789	142	67	264	447	869

### Phylogeny and distribution

**Fig 3:**
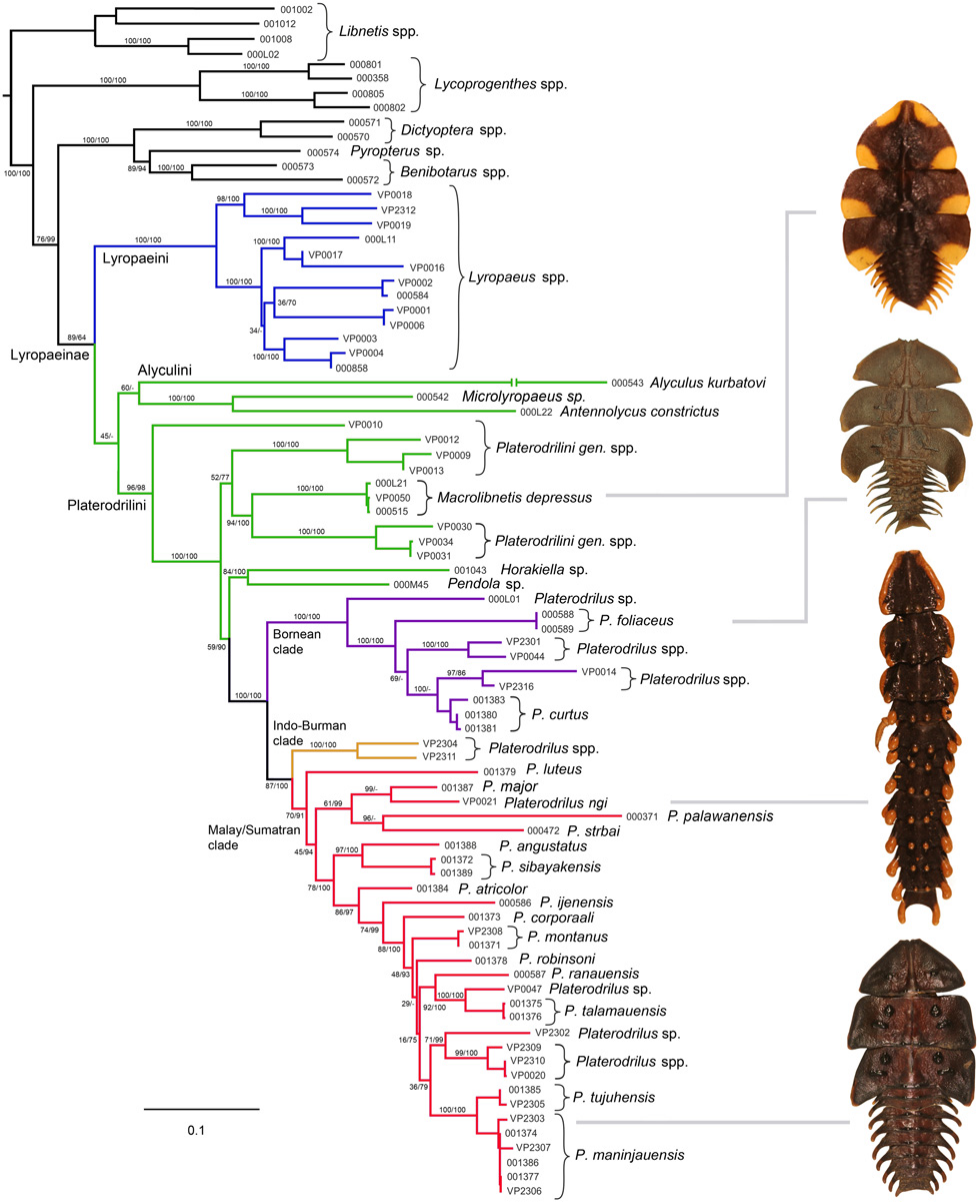
Phylogenetic hypothesis for *Platerodrilus* Pic, 1921 based on a maximum likelihood analysis of the Muscle alignment. Numbers at the branches are maximum likelihood bootstrap values and Bayesian posterior probabilities.

**Fig 4:**
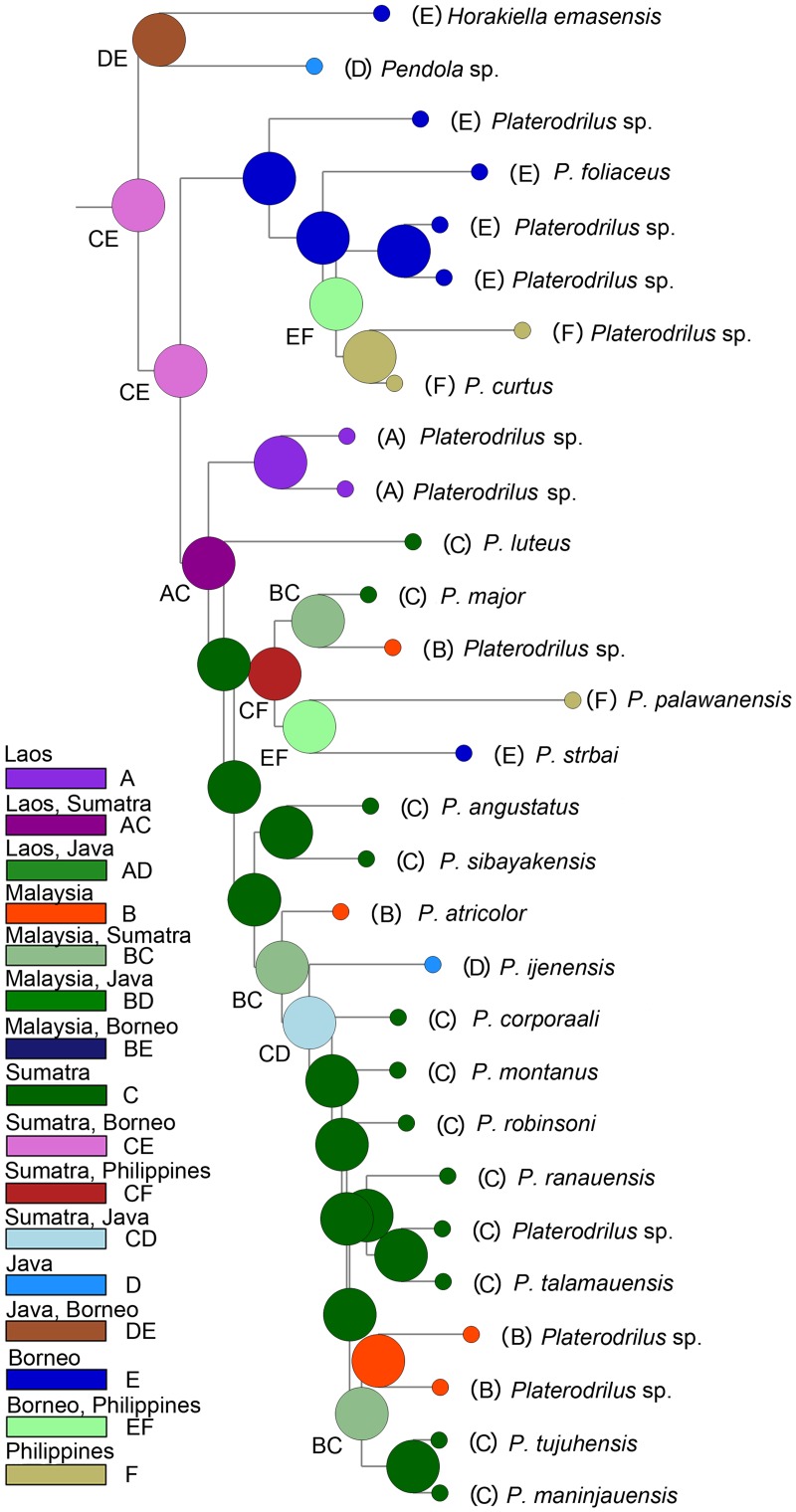
Reconstruction of ancestral ranges using statistical parsimony.

**Fig 5:**
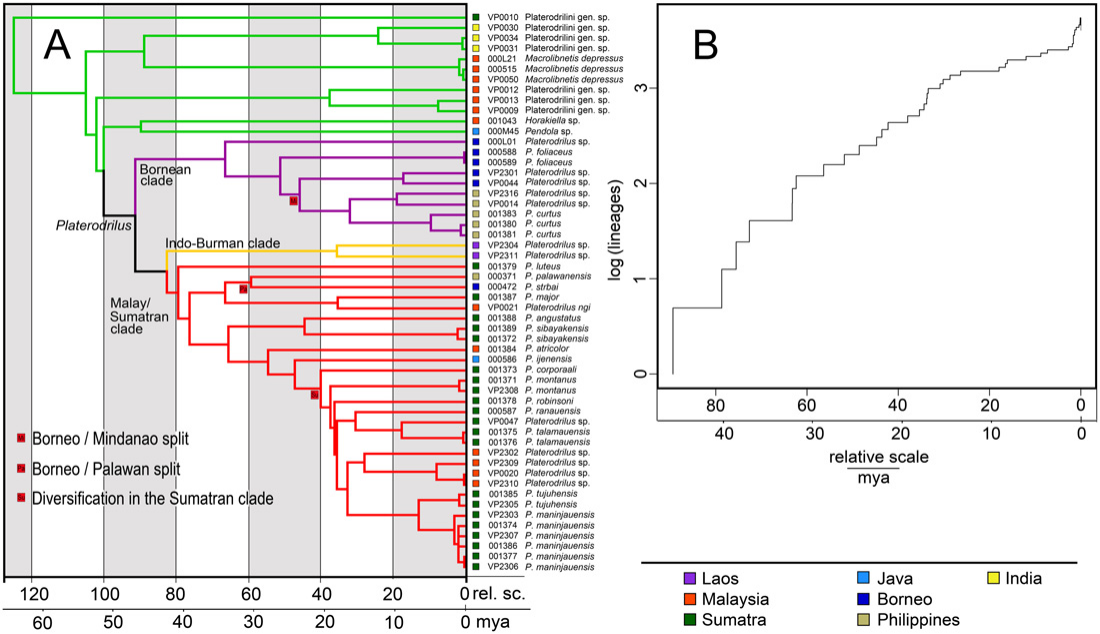
A—Relative age of nodes estimated using penalized likelihood. The age of *Platerodrilus* was arbitrarily set to 100. The lower axes show ageestimation in mya derived from the age of *Platerodrilus* inferred at 47 mya by Bocak *et al*. (2008). B—Lineage-through-time pot for *Platerodrilus*, the time axis is calibrated as in Fig 5A.

**Fig 6:**
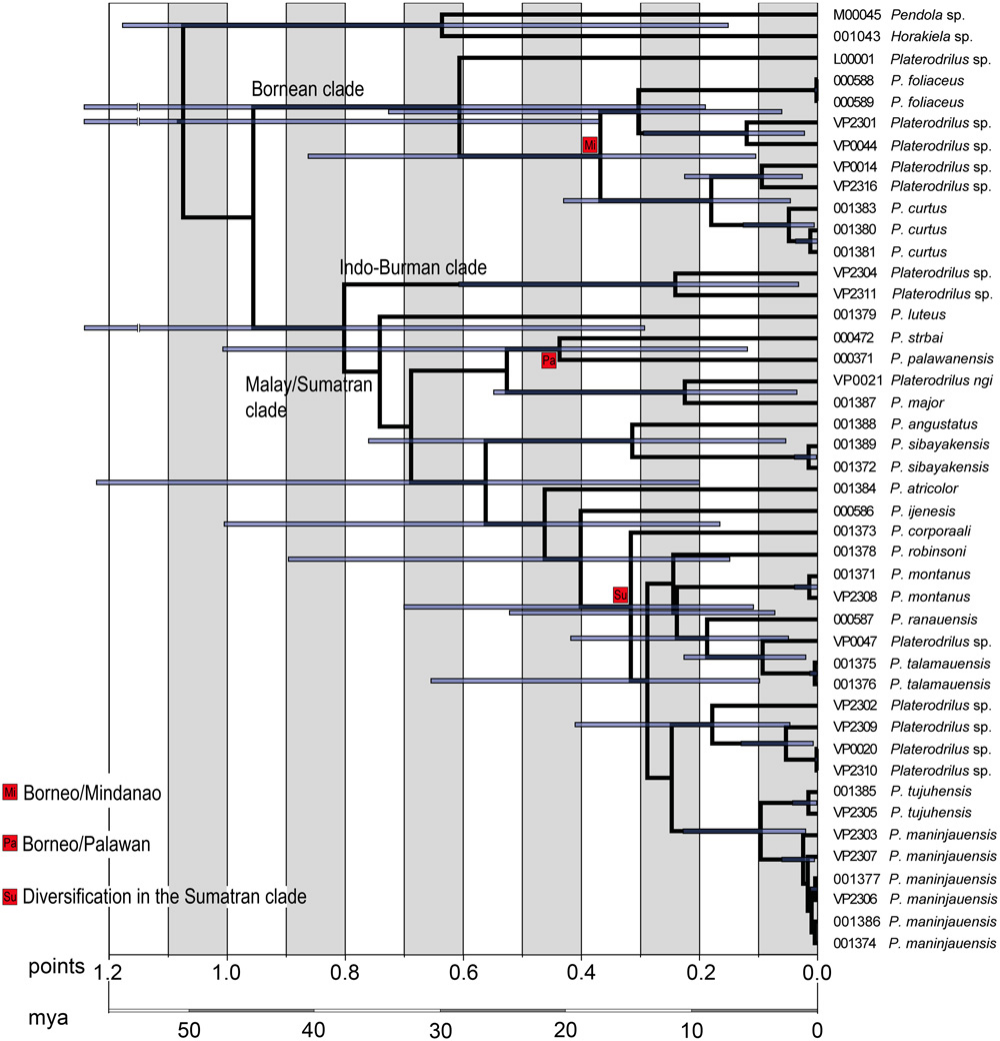
Timing of the *Platerodrilus* radiation. Estimated ages of nodes are based on Bayesian analysis of all fragments and Muscle alignment. The bars depict 95% confidence intervals.

The topologies produced from BlastAlign, Muscle and Clustal alignments analyzed under maximum likelihood method (ML) and Bayesian inference (BI) were similar (Fig 3, S1A–S1C Fig). The Platerodrilini were regularly inferred as monophyletic group with robust support (BS 96%, PP 98%). Only the ML analysis of the Muscle alignment did not recover Platerodini, when *Alyculus* + *Microlyropaeus* + *Antennolycus* were found as a sister to remaining Lyropaeinae. *Macrolibnetis*, *Horakiella*, *Pendola* and related genera represented the basal grade within Platerodrilini (Fig 3) and *Platerodrilus* clade was consistently recovered as a subordinate branch within Platerodrilini (BS and PP 100%). *Platerodrilus* split in a Bornean clade (BS and PP 100%), Indo-Burman clade and (BS and PP 100%) and Malay/Sumatran clade (Fig 1; BS 87%, PP 100%). The alternative topologies were recovered in the terminal clades: the relationships among Bornean species (*P*. *curtus* and two unidentified spp.) was unstable, the sister species of *P*. *palawanensis* were either *P*. *strbai* or *P*. *ngi* (Fig 3, S1 Fig) and variable relationships were recovered for *P*. *robinsoni* and *P*. *ijenensis* within the Malay/Sumatran clade (Fig 3, S1 Fig). The ancestral ranges were inferred using parsimony and the phylogenetic hypothesis produced by the ML analysis of the Muscle alignment (Fig 3). We identified Sumatra and Borneo as an ancestral range of *Platerodrilus* with available distributional data (Fig 4). Borneo was identified as an ancestral range of the *P*. *foliaceus* and related species from Borneo and Mindanao (i.e., *P*. *foliaceus*, *P*. *curtus* and three unidentified species). This lineage is designated as the Bornean clade in Figs 3, 5, 6 and S1 Fig). Further the basal lineage known from Laos and Thailand in two unidentified female larvae (VP2304, 2311) is designated as the Indo-Burman clade. Most species of *Platerodrilus* are known from Sumatra and we identified the Malay Peninsula and Sumatra as ancestral range for twenty species of *Platerodrilus*. The clade is designated as the Malay/Sumatran in Fig 3 and only two species of this clade are known from Borneo and a single species from Palawan.

Altogether 29 species of 42 formally described were available for DNA analyses and we assign species, which could not be sequenced, to three principal clades using morphology. Accordingly, eight species are assigned to the Bornean clade (five of them sequenced, Fig 3): five from Borneo, two from the Philippines, and one from Sumatra (Fig 2). The Indo-Burman clade (Figs 2, 4 and 5A, voucher numbers VP2304, 2311) is known only in larvae and without adults we cannot confirm the association of the sequenced larvae with any of the previously described three species from the region, therefore these are placed in the clade only tentatively. The Malay/Sumatran clade is represented by 17 spp. in Sumatra, 6 spp. in Java, 4 spp. in Peninsular Malaysia, 2 spp. in Borneo and a single species in Palawan (Figs 2, 3 and 5A). Some species were represented in the sequenced material only by female larvae and could not be identified to the species level. As we suppose, that at least some of them are conspecific with Sumatran and Malay species described in males, we do not list them in Fig 2.

As no fossils are available for calibration, the approximate estimation of absolute time was derived from the previous analysis, which dealt with whole family [1] and dated the basal split of *Platerodrilus* to 47 mya. The relatively ancient origin of Platerodini and *Platerodrilus* is supported by the fact that they represent one of the deep lineages in the phylogeny of net-winged beetles and that they are a morphologically very distinct group [1].

The age estimation of principal splits using penalized likelihood shows that *Platerodrilus* originated early in evolution of the Platerodrilini (Fig 5A) and the principal clades within *Platerodrilus* were established soon thereafter (Fig 5A). With the basal split in *Platerodrilus* dated to 47 mya, the principal clades separated ~40 mya, the species from Borneo and Palawan or Mindanao split ~25 mya and the multiple splits giving origins of Sumatran clades are dated at ~17 mya. The ltt plot (Fig 5B) suggests increased diversification rate at the beginning of *Platerodrilus* evolution and at ~17 mya. The diversification slowed down since ~12 mya to present.

Alternative dating using the Bayesian approach provided similar, only slightly shallower time estimation for important splits in *Platerodrilus* (Fig 6). The analysis was affected by the missing fragments and the 95% confidence intervals were large. Nevertheless, we can conclude that basal splits between lineages from Indo-Burma, Borneo and the Malay Peninsula are ancient and dated to ~35 mya, the splits Palawan or Mindanao species are dated to ~20 mya and the beginning of the radiation in the Sumatran clade to ~15 mya. There were not identified any dispersal events between major geographic regions during the Pliocene and Pleistocene low sea stands in the last 3 million years even when the uncertainty of the dating is considered (Figs 3 and 5A).

## Discussion

The current dataset presented here provides more data for study of relationships of neotenic net-winged beetle lineages than the previous study, which included only four individuals [1]. The phylogenetic analyses confirm that Lyropaeinae represent a monophylum, which consists of Lyropaeini, Alyculini and Platerodrilini, all of them with confirmed or previously proposed neotenic development of females. Further, all inferred topologies indicate that the large-bodied larviform females evolved independently in both lineages: in *Lyropaeus* Waterhouse, 1878 (*sensu* Masek *et al*. [2]) in Lyropaeini and in *Platerodrilus* and *Macrolibnetis* (Platerodrilini). The first larviform platerodriline females are reported from Northeastern India and they represent a sister clade to *Macrolibnetis depressus* Pic, 1938 from Peninsular Malaysia in the present analyses. The available taxa from India are known only in the female larvae (terminals VP0030, 31, 34, Fig 3) and further information is needed before their placement in *Macrolibnetis* is confirmed. The adults of further genera, *i*.*e*., *Horakiella*, *Pendola*, *Microlyropaeus*, *Antennolycus*, and several undescribed taxa (Fig 1), are exclusively known in adult males, when about two hundred specimens are currently available in collections. Both the larvae and adult females of these genera remain unknown and their neoteny has been only inferred from the absence of females in collected samples, morphology of males (all males are small bodied, with reduced mouthparts and often with light coloured and weakly sclerotized apexes of antennae) and proven presence of neotenic females in related genera (Fig 3). Field research in South East Asia yielded several dozen large-bodied larvae, all morphologically distinct samples were sequenced and all of them represent various species of *Platerodrilus* and *Macrolibnetis*. Neotenic female development has already been reported for a few species of these genera: *Platerodrilus paradoxus* [4], *P*. *ruficollis* (Pic, 1942) (= *Duliticola hoiseni* Wong, 1996) and *Macrolibnetis depressus* Pic, 1928 [4, 5, 7]. Therefore, considering the high number of identified males and females exclusively in *Platerodrilus*, we suppose that the adult larviform females of other genera are of a similar body size to males and that given their small size they have been overlooked until now.

### Phylogeography and diversification of *Platerodrilus*

The distribution of *Platerodrilus* is limited to the eastern part of the Oriental region (Fig 2). The highest diversity of *Platerodrilus* is known from Sumatra (18 spp.), Borneo (7 spp.) and Peninsular Malaysia (5 spp.). Only a few species have been reported from Vietnam, Laos, Thailand and southernmost China ([21] and unpublished records from Hainan and Yunnan; Yun Li, pers. comm.). A similarly limited number of species is known from the Philippines (Fig 2). Despite presumed ancient origin of *Platerodrilus* supported by morphological and genetic divergence [1], the range of these neotenic beetles is limited to regions with stable occurrence of humid tropical forests and they are not able to readily expand their ranges during periods with favourable climatic conditions. Although other lycid lineages dispersed from the Oriental region to northern China and Japan in a high number of species, *e*.*g*., Metriorrhynchini [22], *Platerodrilus* have their northernmost limits in Hainan and southern and south-western Yunnan.

The phylogeographic analysis suggests Borneo and Sumatra as an ancestral range of *Platerodrilus* (Fig 3); *P*. *foliaceus* and its relatives identified on the basis of similar robust parameres occur predominantly in Borneo and *P*. *sinuatus* and its relatives, characterized by the slender curved phallus and slender parameres, in Sumatra and continental Asia [3]. Three principal splits in the phylogeny of *Platerodrilus* were designated as the Bornean (+Philippines), Malay/Sumatran (+ Java, Borneo, Palawan) and Indo-Burman clades (Figs 1 and 3). The ranges and centres of diversity of these clades differ.

The Bornean clade contains five Bornean, two Philippine species and only a single species of the clade occurs in Sumatra (*P*. *wongi* from Northern Sumatra, not available for DNA analysis and assigned to the clade using morphology). The Philippine species represent a terminal lineage and a single colonization event to the Philippines was recovered in an early phase of diversification of the clade. The dating must be viewed as preliminary, nevertheless the approximate estimation of their age points to a similar age as recovered in Philippine *Scarelus* by Malohlava and Bocak [23], when mutation rate was used for dating in this distantly related lineage. The inferred age of ~25 my is close to the glacial maximum in the early Oligocene [24] and tectonic activity of the Sulu-Cagayan Arc [20]. The neotenics are not able to disperse across the sea and the indication of Oligocene colonization of the terrains forming the present day Philippines suggest a dry-land connection with Borneo. The dependence on a land connection for dispersal is supported by the fact that neither *Scarelus* nor *Platerodrilus* were able to disperse to the Philippines in the Pleistocene when most of the shelf was subaerial and the overseas distances lower than now [25]. Similarly, other neotenics are missing even in close regions if these are isolated by sea (*e*.*g*., the absence of neotenic leptolycines in Jamaica and other Caribbean islands [26]).

The Malay/Sumatran clade was represented in the current molecular analysis by 17 species. The ancestral range was identified in Sumatra (Fig 4) and most species never dispersed outside of the eastern part of the Sunda Shelf, *i*.*e*., Sumatra, Java and Malaya. Only *P*. *borneensis* Wittmer, 1966 (unavailable for DNA isolation), *P*. *strbai* Kazantsev, 2009 and *P*. *palawanensis* Masek et Bocak, 2014 (Fig 3) occur in Borneo and Palawan. The molecular phylogeny supports an early origin of *P*. *palawanensis* (30 mya and 20 mya in penalized likelihood and Bayesian analyses, respectively) and no exchange between faunas of Borneo, Malaya and Sumatra during Pliocene and Pleistocene low sea levels [27, 28]. The geographically close, repeatedly connected terrains of Sumatra and the Malay Peninsula also hold different fauna at the species level and the number of dispersal between them is limited. The single dispersal from Sumatra to the Malay Peninsula was dated to ~18 or ~12 mya according to available analyses (Figs 5A and 6). The other identified species pairs occurring in these regions had split in the Upper Oligocene and Lower Miocene (*Platerodrilus ngi* and *P*. *major*, 17mya), *P*. *luteus* represents an ancient lineage in the Malay/Sumatran clade and *P*. *angustatus* + *P*. *sibayakensis* clade separated from their relatives ~33 or 26 mya (Figs 5A and 6). We did not recover any more recent dispersal events (Figs 5A and 6). The fauna of Java was represented by a single species *P*. *ijenensis* Masek et Bocak, 2014 and this species was recovered as a sister to the diversified crown clade of Sumatran and Malay species. The Sumatran fauna is the only one, which is represented by a sufficient number of lineages to estimate the dynamics of their diversification. The ltt plot (Fig 5B) shows the increased diversification rate in *Platerodrilus* at ~17 mya and subsequent slow down till present. Alternatively, this sequence of splits was dated to 15 mya using the Bayesian inference (Fig 6). We suppose that the emergence of Sumatra 15 mya [20] and formation of the Barisan mountain range triggered diversification of *Platerodrilus*. The stable rainforest conditions in those areas isolated during glacial minima resulted in populations with lower diversification rates in the last 10 my. Dense sampling of species and populations is needed for a comprehensive analysis of speciation rates and these results must be viewed as preliminary.

The low dispersal propensity favours in situ diversification and very limited exchange between major geographical regions. No *Platerodrilus* species has been found in more than a single island and/or a mountain region. The long-term sequence of connection and isolation periods in Southeast Asia during the last 2.7 my [29] provided favourable conditions for multiple dispersal events at least in the last several million years, but no dispersal events were identified in *Platerodrilus*. The reasons for observed patterns include the above mentioned low dispersal propensity, but also the climatic conditions in the subaerial Sunda Shelf. The rainforests in South East Asia depend on the monsoon system, which developed after the uplift of the Himalayas, but these rich habitats covered a variable part of the shelf depending on climatic fluctuations. The cool periods resulted in aridisation of the interior of the shelf [30] and the savannah regions might have prevented the exchange between centres of diversity in eastern and western mountain ranges, which obtained a higher amount of rainfall and house very diverse fauna of neotenics. Similarly, decreased diversity of termites was reported in previously arid regions by Gathorne-Hardy *et al*. [31], but the preserved distribution patterns evolved in the described case during recent Pleistocene periods of climatic fluctuations. In contrast, the faunas of *Platerodrilus* remained isolated in the ancestral ranges over much longer time span and we identified only several dispersal events and/or vicariance between these centres of diversity in the Miocene or earlier.

The low dispersal propensity of neotenic net-winged beetles also affects diversification within individual islands. We found the complete turn over with no species overlap between mountain ranges in Sumatra (Fig 2), from where the higher density of sampling was available. Altogether 18 species occur in Sumatra and most are closely related and belong to the terminal lineage designated as the Malay/Sumatran clade (Fig 5A). The individual mountain systems house completely distinct faunas and no species was collected in two or more distant localities. The stability of the tropical forests since the uplift of the island is postulated to be a necessary condition for *in situ* evolution and long-term persistence of *Platerodrilus* in Sumatra. Additionally, the extremely low dispersal propensity of larviform females contributes to the observed small ranges and the whole-year humid mountain forests in the Sumatran mountain massifs represent separate "islands" where populations of *Platerodrilus* diversified in isolation. The protection of major forest complexes like the Leuser National Park is important, but our findings suggest that only a fragment of genetic diversity is housed even in such a large national park. If diversity of the organisms with limited dispersal propensity is to be protected, the preservation of rainforest habitats is needed in a form of an extensive network covering most of the mountain ranges in South East Asia.

## Supporting Information

S1 Fig The Bayesian phylogenetic trees of *Platerodrilus* inferred from the dataset aligned using (A) BlastAlign, (B) Clustal and (C) Muscle. (TIF)

S1 Table Taxonomic coverage, geographic origin, and GenBank accession numbers. (PDF)

S2 Table The locality data for *Platerodrilus* samples. (PDF)

S3 Table Primers used for PCR amplifications. (PDF)
