# Supplementary figures and images for: Molecular Phylogeny Reveals High Diversity, Geographic Structure and Limited Ranges in Neotenic Net-Winged Beetles Platerodrilus (Coleoptera: Lycidae)
Source: PLoS One. 2015 Apr 28;10(4):e0123855. doi: 10.1371/journal.pone.0123855 (PMC4412711; doi:10.1371/journal.pone.0123855)

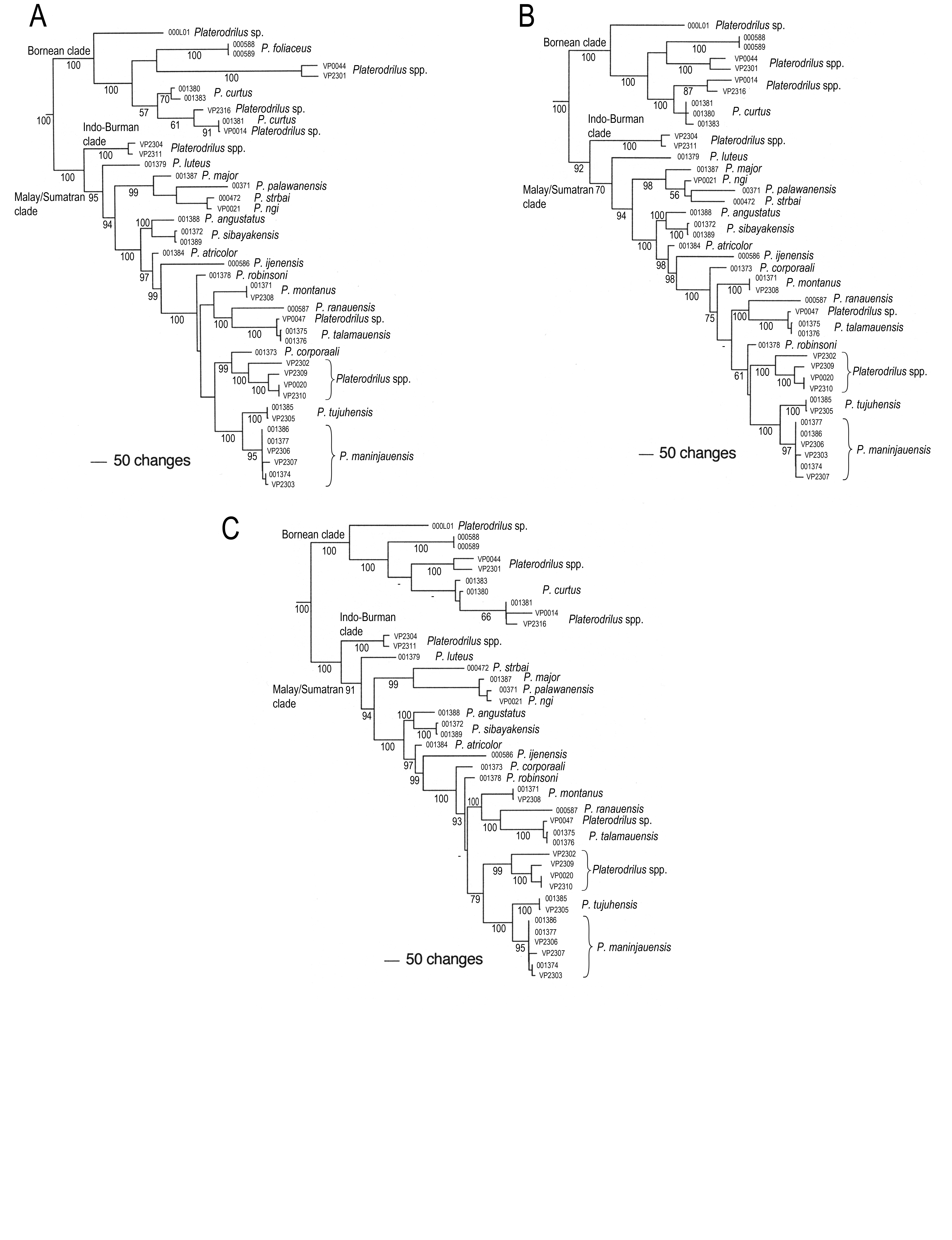

Supplement: S1 Fig — (TIF) [file pone.0123855.s001.tif]
