# Supplementary material for: Molecular Phylogeny Reveals High Diversity, Geographic Structure and Limited Ranges in Neotenic Net-Winged Beetles Platerodrilus (Coleoptera: Lycidae)
Source: PLoS One. 2015 Apr 28;10(4):e0123855. doi: 10.1371/journal.pone.0123855 (PMC4412711; doi:10.1371/journal.pone.0123855)
Supplement: S1 Table — (PDF) [file pone.0123855.s002.pdf]

Species	Voucher UPOL+	Locality data	18S	28S	16S	COI	ND5
Alyculus kurbatovi	000543	Java	DQ181072	DQ181146	DQ180998	DQ181220	DQ181374
Antenolycus constrictus	000L22	Malaysia	DQ181051	DQ181125	DQ180977	DQ181199	DQ181353
Benibotarus nigripennis	000572	Japan	DQ181075	DQ181149	DQ181001	DQ181223	DQ181377
Benibotarus spinicoxis	000573	Japan	DQ181076	DQ181150	DQ181002	DQ181224	DQ181378
Dictyoptera elegans	000570	Japan	DQ181073	DQ181147	DQ180999	DQ181221	DQ181375
Dictyoptera speciosa	000571	Japan	DQ181074	DQ181148	DQ181000	DQ181222	DQ181376
Horakiella emasensis	001043	Malaysia	DQ181110	DQ181184	DQ181036	DQ181258	DQ181412
Libnetis granicollis	001012	Japan	DQ181107	DQ181181	DQ181033	DQ181255	DQ181409
Libnetis sp.	001002	Sumatra	DQ181104	DQ181178	DQ181030	DQ181252	DQ181406
Libnetis sp.	001008	Malaysia	DQ181105	DQ181179	DQ181031	DQ181253	DQ181407
Libnetis sp.	000L02	Sabah	DQ181038	DQ181112	DQ180964	DQ181186	DQ181340
Lycoprogenthes sp.	000801	Sumatra	DQ181095	DQ181169	DQ181021	DQ181243	DQ181397
Lycoprogenthes sp.	000802	Java	DQ181096	DQ181170	DQ181022	DQ181244	DQ181398
Lycoprogenthes sp.	000805	Sumatra	DQ181097	DQ181171	DQ181023	DQ181245	DQ181399
Lycoprogenthes sp.	000358	Java	DQ181070	DQ181144	DQ180996	DQ181218	DQ181372
Lyropaeus sp.	VP0016	India	KC736893	KC736902	KC736885	KC736912	KC736921
Lyropaeus sp.	VP0017	India	KC736894	KC736903	KC736886	–	–
Lyropaeus sp.	VP2312	India	KC736897	KC736906	KC736887	KC736915	KC736924
Lyropaeus dominator	VP0003	Malaysia	KC736890	KC736899	KC736882	KC736909	KC736918
Lyropaeus optabilis	VP0004	Malaysia	KC736891	KC736900	KC736883	KC736910	KC736919
Lyroneces optabilis	000585	Malaysia	DQ181088	DQ181162	DQ181014	DQ181236	DQ181390
Lyropaeus philippinensis	VP0018	Philippines	KC736895	KC736904	–	KC736913	KC736922
Lyropaeus philippinensis	VP0019	Philippines	KC736896	KC736905	–	KC736914	KC736923
Lyropaeus ritsemae	VP0001	Sumatra	KC736888	KC736898	KC736880	KC736907	KC736916
Lyropaeus ritsemae	VP0006	Sumatra	KC736892	KC736901	KC736884	KC736911	KC736920
Lyropaeus rubrostriatus	000L11	Malaysia	DQ181042	DQ181116	DQ180968	DQ181190	DQ181344
Lyropaeus waterhousei	VP0002	Sumatra	KC736889	–	KC736881	KC736908	KC736917
Lyropaeus waterhousei	000584	Sumatra	DQ181087	DQ181161	DQ181013	DQ181235	DQ181389
Macrolibnetis depressus	000515	Malaysia	–	–	–	FJ390411	FJ390413
Macrolibnetis depressus	VP0050	Malaysia	KF802504	KF802506	KF802467	–	KF802538

Macrolibnetis depressus	000L21	Malaysia	DQ181050	DQ181124	DQ180976	DQ181198	DQ181352
Microlyropaeus dembickyi	000542	Sumatra	DQ181071	DQ181145	DQ180997	DQ181219	DQ181373
Pendola sp.	000M45	Java	DQ181058	DQ181132	DQ180984	DQ181206	DQ181360
Platerodrilini gen. sp.	VP0009	Malaysia	KF802497	KF802507	KF802457	KF802527	KF802539
Platerodrilini gen. sp.	VP0010	Sumatra	KF802498	KF802508	KF802480	KF802533	KF802542
Platerodrilini gen. sp.	VP0012	Malaysia	KF802499	KF802509	KF802458	KF802528	KF802541
Platerodrilini gen. sp.	VP0013	Malaysia	KF802500	KF802510	–	KF802529	KF802540
Platerodrilini gen. sp.	VP0030	India	KF802493	KF802511	KF802462	KF802530	KF802543
Platerodrilini gen. sp.	VP0031	India	KF802495	KF802512	KF802463	KF802531	–
Platerodrilini gen. sp.	VP0034	India	KF802496	KF802513	KF802464	KF802532	KF802544
Platerodrilus angustatus	001388	Sumatra	KF625713	KF626299	KF626001	KF625406	–
P. atricolor	001384	Malaysia	KF625710	KF626296	–	–	–
P. corporaali	001373	Sumatra	KF625702	KF626288	KF625991	KF625400	–
P. curtus	001380	Philippines	KF625708	KF626294	KF625997	KF625404	–
P. curtus	001381	Philippines	KF625781	KF626365	KF626073	–	–
P. curtus	001383	Philippines	KF625782	KF626366	KF626074	KF625474	–
P. foliaceus	000588	Borneo	DQ181091	DQ181165	DQ181017	DQ181239	DQ181393
P. foliaceus	000589	Borneo	–	–	EF143214	EF143229	EF143243
P. ijenensis	000586	Java	DQ181089	DQ181163	DQ181015	DQ181237	DQ181391
P. luteus	001379	Sumatra	KF625707	KF626293	KF625996	KF625403	–
P. major	001387	Sumatra	KF625712	KF626298	KF626000	–	–
P. maninjauensis	001374	Sumatra	KF625703	KF626289	KF625992	KF625401	–
P. maninjauensis	001377	Sumatra	KF625705	KF626291	KF625994	–	–
P. maninjauensis	001386	Sumatra	–	KF625783	KF626075	–	–
P. maninjauensis	VP2303	Sumatra	KF802485	KF802514	KF802470	–	KF802545
P. maninjauensis	VP2306	Sumatra	KF802487	KF802515	KF802473	–	KF802546
P. maninjauensis	VP2307	Sumatra	KF802488	KF802516	KF802474	–	KF802547
P. montanus	001371	Sumatra	KF625700	KF626286	KF625989	KF625398	–
P. montanus	VP2308	Sumatra	KF802489	KF802517	KF802475	–	KF802549
Platerodrilus ngi	VP0021	Singapore	KF802481	KF802520	KF802461	–	–
P. palawanensis	000371	Philippines	–	–	–	–	KF802552
P. ranauensis	000587	Sumatra	DQ181090	DQ181164	DQ181016	DQ181238	DQ181392
P. robinsoni	001378	Sumatra	KF625706	KF626292	KF625995	–	–
P. sibayakensis	001372	Sumatra	KF625701	KF626287	KF625990	KF625399	–
P. sibayakensis	001389	Sumatra	KF625784	KF626367	–	KF625475	–

Platerodrilus sp.	000L01	Sabah	DQ181037	DQ181111	DQ180963	DQ181185	DQ181339
Platerodrilus sp.	VP0044	Sabah	KF802505	–	KF802465	–	KF802550
Platerodrilus sp.	VP2301	Sabah	KF802483	KF802518	KF802468	–	KF802551
Platerodrilus sp.	VP0014	Philippines	KF802482	KF802519	KF802459	KF802534	KF802553
Platerodrilus sp.	VP0020	Malaysia	KF802554	–	KF802460	KF802535	KF802554
Platerodrilus sp.	VP0047	Sumatra	KF802502	–	KF802466	–	KF802555
Platerodrilus sp.	VP2302	Malaysia	KF802484	KF802521	KF802469	–	KF802556
Platerodrilus sp.	VP2304	Thailand	KF802486	KF802522	KF802471	–	–
Platerodrilus sp.	VP2309	Malaysia	KF802490	KF802524	KF802476	–	KF802557
Platerodrilus sp.	VP2310	Malaysia	KF802491	KF802525	KF802477	KF802536	KF802558
Platerodrilus sp.	VP2311	Laos	KF802503	–	KF802478	–	KF802559
Platerodrilus sp.	VP2316	Philippines	KF802492	KF802526	KF802479	–	KF802560
P. strbai	000472	Sabah	–	–	–	KF802537	KF802561
P. talamauensis	001375	Sumatra	KF625780	KF626364	KF626072	KF625473	–
P. talamauensis	001376	Sumatra	KF625704	KF626290	KF625993	KF625402	–
P. tujuhensis	001385	Sumatra	KF625711	KF626297	KF625999	KF625405	–
P. tujuhensis	VP2305	Sumatra	KF802494	KF802523	KF802472	–	KF802548
Pyropterus nigroruber	000574	Japan	DQ181077	DQ181151	DQ181003	DQ181225	DQ181379

Note. The *rrnL* fragments with KF Genbank accession codes were published by Masek and Bocak (2014) and fragments with DQ codes by Bocak and Bocakova (2008).
