# Supplementary material for: Molecular Phylogeny Reveals High Diversity, Geographic Structure and Limited Ranges in Neotenic Net-Winged Beetles Platerodrilus (Coleoptera: Lycidae)
Source: PLoS One. 2015 Apr 28;10(4):e0123855. doi: 10.1371/journal.pone.0123855 (PMC4412711; doi:10.1371/journal.pone.0123855)
Supplement: S2 Table — (PDF) [file pone.0123855.s003.pdf]

Genus Species	Voucher Number	Area	Locality	Latitude	Longitude
Platerodrilus Pic,					
P. angustatus	001388	Sumatra	Sumatra Utara, Brastagi env.	3° 13'N	98° 30'E
P. atricolor	001384	Malay Peninsula	Pahang, Cameron High.	4° 16'N	101° 19'E
P. corporaali	001373	Sumatra	Sumatra Barat, Bukittinggi env.	0° 23'S	100° 26'E
P. curtus	001380	Mindanao	Bukidnon, Maramag env.	0° 55'N	124° 54'E
P. curtus	001381	Mindanao	Bukidnon, Maramag env.	7° 55'N	124° 54'E
P. curtus	001383	Mindanao	Bukidnon, Maramag env.	7° 55'N	124° 54'E
P. foliaceus	000588	Borneo	Kalimantan, Muara Teweh	0° 54'N	114° 55'E
P. foliaceus	000589	Borneo	Kalimantan, Muara Teweh	0° 54'N	114° 55'E
P. ijenensis	000586	Java	Java Timur, Ijen Plateau	8° 05'S	114 °17'E
P. luteus	001379	Sumatra	Jambi, Gunung Kerinci	1° 45'S	101° 25'E
P. major	001387	Sumatra	Sumatra Utara, Brastagi env.	3° 13'N	98° 30'E
P. maninjauensis	001374	Sumatra	Sumatra Barat, Danau Maninjau	0° 20'S	100° 14'E
P. maninjauensis	001377	Sumatra	Sumatra Barat, Danau Maninjau	0° 20'S	100° 14'E
P. maninjauensis	001386	Sumatra	Sumatra Barat, Danau Maninjau	0° 20'S	100° 14'E
P. maninjauensis	VP2303	Sumatra	Sumatra Barat, Gunung Talamau	0° 03'N	99° 57'E
P. maninjauensis	VP2306	Sumatra	Sumatra Barat, Danau Maninjau	0° 20'S	100° 14'E
P. maninjauensis	VP2307	Sumatra	Sumatra Barat, Danau Maninjau	0° 20'S	100° 14'E
P. montanus	001371	Sumatra	Sumatra Utara, Brastagi env.	3° 13'N	98° 30'E
P. montanus	VP2308	Sumatra	Sumatra Utara, Brastagi env.	3° 11'N	98° 23'E
Platerodrilus ngi	VP0021	Singapore	Singapore	1° 23'N	103° 48'E
P. palawanensis	000371	Palawan	Cleopatra Needle	10° 03'N	118 °58'E
P. ranauensis	000587	Sumatra	Sumatra Selatan, Danau Ranau	4° 54'S	103° 58'E
P. robinsoni	001378	Sumatra	Jambi, Gunung Kerinci	1° 45'S	101° 25'E
P. sibayakensis	001372	Sumatra	Sumatra Utara, Brastagi env.	3° 13'N	98° 30'E
P. sibayakensis	001389	Sumatra	Sumatra Utara, Brastagi env.	3° 13'N	98° 30'E
Platerodrilus sp.	000L01	Borneo	Sabah, Gn Emas	5° 49'N	116° 19'E
Platerodrilus sp.	VP0044	Borneo	Sabah, Gn Emas	5° 49'N	116° 19'E
Platerodrilus sp.	VP2301	Borneo	Sabah, Gn Emas	5° 49'N	116° 19'E

Platerodrilus sp.	VP0014	Mindanao	Davao Prov., Panabo env.	7° 20'N	125° 45'E
Platerodrilus sp.	VP0020	Malaysia	Pahang, Cameron High.	4° 22'N	101° 20'E
Platerodrilus sp.	VP0047	Sumatra	Sumatra Barat, Bukittnggi env.	0° 23'S	100 26'E
Platerodrilus sp.	VP2302	Malaysia	Johor, Kotatinggi env.	1° 49'N	103° 57'E
Platerodrilus sp.	VP2304	Thailand	Nan province, Doi Phuka N. P.	19° 12'N	101° 05'E
Platerodrilus sp.	VP2309	Malay Peninsula	Perak, Taiping env.	4° 50'N	100° 51'E
Platerodrilus sp.	VP2310	Malay Peninsula	Pahang, Cameron High.	4° 16'N	101° 19'E
Platerodrilus sp.	VP2311	Laos	Hua Phan Prov., Hua Phan env.	20° 12'N	104° 01'E
Platerodrilus sp.	VP2316	Mindanao	Davao prov., Panabo env.	7° 20'N	125° 45'E
P. strbai	000472	Borneo	Sabah, Gn Emas	5° 49'N	116° 19'E
P. talamauensis	001375	Sumatra	Sumatra Barat, Gunung Talamau	0° 03'S	99° 57'E
P. talamauensis	001376	Sumatra	Sumatra Barat, Gunung Talamau	0° 03'S	99° 57'E
P. tujuhensis	001385	Sumatra	Jambi, Gunung Kerinci	1° 45'S	101° 25'E
P. tujuhensis	VP2305	Sumatra	Jambi, Gunung Kerinci	1° 45'S	101° 25'E
