# Supplementary material for: Molecular Phylogeny Reveals High Diversity, Geographic Structure and Limited Ranges in Neotenic Net-Winged Beetles Platerodrilus (Coleoptera: Lycidae)
Source: PLoS One. 2015 Apr 28;10(4):e0123855. doi: 10.1371/journal.pone.0123855 (PMC4412711; doi:10.1371/journal.pone.0123855)
Supplement: S3 Table — (PDF) [file pone.0123855.s004.pdf]

Fragment	Code -	mer	Sequence (5’ >> 3’)
18S rRNA	5’	24	GACAACCTGGTTGATCCTGCCAGT
	b5.0	19	TAACCGCAACAACTTTAAT
	ai	22	CCTGAGAAACGGCTACCACATC
	b2.5	20	TCTTTGGCAAATGCTTTCGC
	a1.0	20	GGTGAAATTCTTGGACCGTC
	bi	20	GAGTCTCGTTCGTTATCGGA
	3’I	24	CACCTACGGAAACCTTGTTACGAC
	a2.0	19	ATGGTTGCAAAGCTGAAAC
28S rRNA	ff	20	TTACACACTCCTTAGCGGAT
	dd	19	GGGACCCGTCTTGAAACAC
16S rDNA	16a	20	CGCCTGTTTAACAAAAACAT
	16b	22	CCGGTCTGAACTCAGATCATGT
	ND1A	27	GGTCCCTTACGAATTTGAATATATCCT
cox1 mtDNA	JerM	23	CAACAYYTATTTTGRTTYTTTGG
	Pat	25	TCCATTGCACTAATCTGCCATATTA
	Marilyn	21	TCATAAGTTCAGTATCATTG
	Marcy	27	TARTTCRTATGWTCAATAYCAYTGRTG
nad5 mtDNA	OF1	29	CCTACTCCTGTTTCTGCTTTAGTTCATTC
	R6	29	GAAACGAAAAATCGTATTTAATTTCGACT
